# Language- and Activity-Specific Associations Between English and Chinese Home Literacy Activities and Receptive Vocabulary Among Chinese–Canadian Children: A Repeated Cross-Sectional Study

**DOI:** 10.3390/bs16091592

**Published:** 2026-09-07

**Authors:** Guofang Li, Fubiao Zhen

**Affiliations:** 1 Department of Language and Literacy Education, University of British Columbia, Vancouver, BC V6T 1Z2, Canada; guofang.li@ubc.ca; 2 School of Foreign Studies, South China Normal University, Guangzhou 510631, China

**Keywords:** bilingual home literacy activity, receptive vocabulary, Chinese–English bilingual children

## Abstract

This repeated cross-sectional study examined how English and Chinese home literacy activities were associated with receptive vocabulary among Chinese–English bilingual children in British Columbia, Canada. The study included 123 children in Grade 1 and 126 children in Grade 2. Children’s English and Chinese receptive vocabulary was assessed using standardized vocabulary measures, and parents reported the frequency of five home literacy activities in each language: book or magazine reading, computer or laptop use, television or movie watching, storytelling, and singing songs. Descriptive analyses showed that English home literacy activities were generally more frequent than Chinese activities across both grades. However, correlation and hierarchical regression results showed that Chinese receptive vocabulary was more strongly and more broadly associated with Chinese home literacy activities. In Grade 1, Chinese book or magazine reading was significantly associated with Chinese receptive vocabulary, while English singing songs was significantly associated with English receptive vocabulary. In Grade 2, Chinese book or magazine reading, computer or laptop use, and storytelling were significantly associated with Chinese receptive vocabulary, whereas English book or magazine reading was the only significant home literacy variable for English receptive vocabulary. These findings highlight the language- and activity-specific nature of bilingual home literacy environments.

## 1. Introduction

The home literacy environment (HLE) has been widely recognized as a foundational determinant of children’s early language and literacy development, with its influence extending beyond the preschool years into the early elementary grades (Burgess et al., 2002; Griffin & Morrison, 1997; Payne et al., 1994; Sénéchal & LeFevre, 2002, 2014; Silinskas et al., 2020; van Bergen et al., 2017). Among language and literacy skills, vocabulary occupies a particularly critical position, as it supports children’s oral language development (Dickinson & Tabors, 2001), reading comprehension (Biemiller, 2003), and broader academic learning (Snow et al., 1998). Within the HLE, different home literacy activities may provide different kinds of language input and therefore contribute to vocabulary development in different ways. Book reading may expose children to print-based and contextualized vocabulary (Su et al., 2026); storytelling may support oral narrative language (Sénéchal & LeFevre, 2002, 2014); and digital literacy activities may offer additional multimodal language exposure (Li et al., 2023). Thus, HLE should not be treated as a single undifferentiated construct, especially when the goal is to understand how particular home literacy activities are associated with children’s vocabulary development.

This issue becomes more complex in bilingual families, where home literacy activities are distributed across more than one language. For bilingual children, the mainstream language and the heritage language are not supported by the same language environment. The mainstream language is likely to be supported by schools, peers, and the wider community, whereas the heritage language may be more closely associated with home-based activities and family interactions (e.g., Duursma et al., 2007; Hammer et al., 2011; Hoff, 2018; Ryan, 2021). Existing bilingual HLE studies have mainly examined language exposure, shared reading, book availability, and overall HLE in relation to bilingual vocabulary development across different contexts (e.g., Goodrich et al., 2021; Li et al., 2025; Mak et al., 2025; Sun et al., 2024). However, less is known about how specific home literacy activities in each language are associated with receptive vocabulary in the corresponding language, particularly during the early elementary years when formal schooling becomes increasingly dominant and may alter the role of HLE across the two languages. Therefore, the present study examines how different English and Chinese home literacy activities, including book reading, storytelling, digital literacy activities, and singing songs, are associated with English and Chinese receptive vocabulary among Chinese–English bilingual children in Grade 1 and Grade 2. By doing so, we aim to clarify how bilingual HLE is organized across languages and activity types in relation to early receptive vocabulary development.

### 1.1. Home Literacy Activities and Early Vocabulary Development

Home literacy environment (HLE) has been widely examined as a key context for children’s early language and literacy development. A large body of research has shown that variations in children’s home literacy experiences are associated with early literacy-related outcomes (e.g., Burgess et al., 2002; Griffin & Morrison, 1997; Niklas & Schneider, 2017; Sénéchal & LeFevre, 2002, 2014). Although HLE has been conceptualized in different ways across studies, it is generally treated as a multidimensional construct that includes home literacy resources, such as books and other print materials (Burgess et al., 2002; Griffin & Morrison, 1997; van Bergen et al., 2015), parental literacy beliefs and expectations (Burgess et al., 2002; Sénéchal & LeFevre, 2002; Sonnenschein et al., 2026), parents’ own literacy practices, such as parents’ reading habits and printed resources (Burgess et al., 2002; Payne et al., 1994; van Bergen et al., 2015), and children’s literacy activities, such as shared reading, direct literacy teaching, and children’s independent reading (Payne et al., 1994; Sénéchal & LeFevre, 2002, 2014; Silinskas et al., 2020). Among the existing conceptualizations of HLE, Sénéchal and LeFevre’s Home Literacy Model is particularly influential because it offers one of the earliest and most widely used classifications of home literacy activities (Sénéchal & LeFevre, 2002, 2014). The model distinguishes between formal and informal literacy practices. Formal literacy practices refer to parents’ direct teaching of code-related skills, such as letters, sounds, and characters, whereas informal literacy practices refer to meaning-focused activities, such as shared reading and storytelling.

While this model remains foundational, recent research has increasingly shown that children’s home literacy experiences may extend beyond the formal–informal distinction, especially in linguistically diverse families. Beyond direct teaching and shared reading, studies have increasingly examined a wider range of literacy activities, including children’s independent reading, literacy-related conversations, and digital literacy practices (Georgiou et al., 2021; Li et al., 2024; Silinskas et al., 2020). These activities often involve multiple literacy processes and do not always fit neatly into either the formal or informal literacy activity categories. Furthermore, as the Home Literacy Model was primarily developed and validated in monolingual contexts, its explanatory power may be limited when applied to bilingual or multilingual families, where literacy activities are not only differentiated by type but also by language (Li et al., 2025). The same activity, such as shared reading or direct literacy instruction, may be conducted in the mainstream language, the heritage language, or both, with potentially different contributions to children’s language and literacy development (Georgiou et al., 2021; Li et al., 2022). These differences make it necessary to examine HLE not only by activity type, but also by the language in which each activity takes place.

### 1.2. Bilingual Home Literacy Environment and Receptive Vocabulary Development

For bilingual children, HLE needs to be examined in relation to each language. Bilingual families are not simply monolingual families with two languages added together. Instead, their home literacy environments reflect the unequal status of the two languages. They may also differ according to parental language proficiency, immigration history, and access to literacy resources (Duursma et al., 2007; Hammer et al., 2003; Hoff, 2018; Paradis, 2011; Ryan, 2021). In English-dominant contexts, English is supported not only at home but also through school, peers, and community. In contrast, the heritage language may be more closely associated with home-based input and family literacy practices.

This has led researchers to move beyond a monolingual HLE toward a bilingual or multilingual perspective. Rather than treating HLE as a single language construct, this approach examines home literacy experiences across different languages used within families (Goodrich et al., 2021; Li et al., 2025; Mak et al., 2025). Evidence from bilingual research suggests that HLE effects are often language-specific (Goodrich et al., 2021; Li & Zhen, 2026; Ryan, 2021). For example, Ryan (2021) found that HLE was associated with French receptive vocabulary among French–English bilingual children but not with English vocabulary. Similarly, Goodrich et al. (2021) reported that home literacy experiences predicted vocabulary growth in both Spanish and English among dual language learners, although the strength of these associations varied according to the language and the type of literacy activities provided at home. Together, these findings suggest that bilingual language and literacy development is best understood by considering literacy practices separately within each language. A single overall measure of the home literacy environment may not fully capture the differences between the two languages.

Studies of bilingual development among Chinese–English children have likewise shown that multiple dimensions of the home literacy environment (HLE) contribute to bilingual language development. For example, some studies have highlighted the importance of the quality of parent–child literacy interactions for children’s heritage-language receptive vocabulary (Chen & Ren, 2019; Cheung et al., 2019), while others have documented the positive associations between home language input and shared reading and children’s heritage-language vocabulary development (Mak et al., 2023; Sun et al., 2024). Recent research further suggests that the nature and strength of these associations may vary across languages and developmental stages. For example, Li et al. (2025) examined Chinese–Canadian children in kindergarten and Grade 1 and found that English HLE was significantly associated with English receptive vocabulary in kindergarten but not in Grade 1, whereas Chinese HLE remained significantly associated with Chinese receptive vocabulary across both grades.

These findings also highlight a limitation of the traditional Home Literacy Model (Sénéchal & LeFevre, 2002, 2014), which conceptualizes HLE primarily within a single-language context. As children enter English-medium schooling, English vocabulary development may become increasingly supported by school-based experiences, whereas heritage-language vocabulary continues to rely more heavily on literacy resources and practices at home. To account for these bilingual developmental processes, Li et al. (2025) proposed a home biliteracy model for bilingual families. Rather than treating HLE as a single literacy context, the model conceptualizes home literacy experiences separately for each language and across multiple dimensions. Specifically, it incorporates print resources (e.g., the number of books available at home), digital access and device use, child-independent literacy activities (e.g., independent reading and use of literacy-related resources), and adult-supported literacy activities (e.g., parent–child shared reading and direct literacy instruction).

In sum, existing research suggests that the influence of the home biliteracy environment is multidimensional, language-specific, and may change across developmental stages. English home literacy activities may occur more frequently because English literacy resources are more readily available and children receive extensive exposure through English-medium schooling. However, greater frequency does not necessarily translate into stronger associations with English language development. By contrast, heritage-language literacy activities, although often less frequent, may play a particularly important role because they provide language input that is less available outside the home. These findings highlight the importance of examining not only the frequency of home literacy activities in each language but also the specific dimensions of HLE that are associated with children’s vocabulary development across languages.

### 1.3. The Present Study

Although existing research has substantially advanced our understanding of bilingual home literacy environments (HLEs), several important gaps remain. First, much of the literature has focused on preschool- and kindergarten-aged children. Less is known about the early elementary years, when children’s exposure to English through formal schooling increases and the contributions of home literacy experiences may diverge across English and Chinese. Second, although recent studies have highlighted the multidimensional nature of bilingual HLE, many have relied on overall HLE measures or a limited number of literacy practices, leaving the contributions of specific activities—such as shared book reading, storytelling, singing songs, television or movie watching, and computer or laptop use—less well understood. Third, despite the growing Chinese Canadian population, relatively little research has examined bilingual HLE among Chinese–English children in Canada, where English serves as the societal language while Mandarin and Cantonese function as major heritage languages.

To address these gaps, the present study examined English and Chinese home literacy activities and receptive vocabulary among Chinese–English bilingual children in Grade 1 and Grade 2. Guided by the home biliteracy model (Li et al., 2025), this study aims to clarify how different dimensions of bilingual HLE are associated with English and Chinese receptive vocabulary during the early years of formal schooling. Three research questions anchored this study:

1. What are the patterns of English and Chinese home literacy activities and receptive vocabulary among Chinese–English bilingual children in Grade 1 and Grade 2?
2. What within- and cross-language bivariate associations exist between English and Chinese home literacy activities and children’s English and Chinese receptive vocabulary in Grades 1 and 2?
3. After controlling for demographic characteristics, which language-specific home literacy activities are uniquely associated with children’s receptive vocabulary in the corresponding language in Grades 1 and 2?

## 2. Materials and Methods

The present study employed a repeated cross-sectional design. The data were drawn from a larger longitudinal project investigating the biliteracy development of Chinese–English bilingual children from Chinese immigrant families in a metropolitan area of British Columbia, Canada. Grade 1 and Grade 2 were selected because they represent two early points in formal schooling, when children’s exposure to English through school becomes increasingly salient. Cross-sectional samples in the two grades were collected in consecutive years. Although there was partial overlap in participants (*n* = 89) across the two samples, the present study did not aim to model within-child developmental change. Instead, the analyses focused on grade-specific associations between home literacy activities and receptive vocabulary in the corresponding language.

### 2.1. Participants

As shown in Table 1, the Grade 1 sample included 123 children, with a mean age of 78.15 months (SD = 3.99). Among them, 60 were boys (48.78%) and 63 were girls (51.22%). Twenty-eight children (22.76%) were from low-SES families, whereas 95 children (77.24%) were from non-low-SES families. In terms of immigration status, 27 children (21.95%) were immigrants, and 96 children (78.05%) were born in Canada.

The Grade 2 sample included 126 children, with a mean age of 89.51 months (SD = 4.61). Of these children, 64 were boys (50.79%) and 62 were girls (49.21%). Thirty-three children (26.19%) were from low-SES families, and 93 children (73.81%) were from non-low-SES families. Regarding immigration status, 34 children (26.98%) were immigrants, and 92 children (73.02%) were born in Canada. Together, the two grade-level samples provided cross-sectional data for examining associations between home literacy activities and receptive vocabulary among Chinese–English bilingual children at two early points of schooling.

### 2.2. Demographic Information

Children’s demographic and family background information was collected through a parent questionnaire adapted from previous home literacy environment studies (Dore et al., 2020; Paradis, 2011; Paradis et al., 2021; Sénéchal & LeFevre, 2002, 2014). One parent of each child completed the questionnaire, which included items on children’s age, gender, immigration status, annual household income, and parents’ educational backgrounds.

Family socioeconomic status (SES) was calculated based on annual household income and parental education, following the Hollingshead Index of Social Status (Hollingshead, 1975). Parents’ highest education level and annual household income were each coded on a six-point scale. The SES score was calculated by multiplying the parental education score by three and the household income score by five, with a possible total score of 48. Families with scores lower than 16 were categorized as low-SES families, whereas those with scores of 16 or above were categorized as non-low-SES families.

### 2.3. Bilingual Home Literacy Activities

Children’s bilingual home literacy activities (Table 2) were measured using the Alberta Language Environment Questionnaire (ALEQ; Paradis et al., 2010). Parents reported how frequently their child engaged in five types of literacy-related activities in English and in Chinese: (1) reading books or magazines, (2) using a computer or laptop, (3) watching television or movies, (4) storytelling, and (5) singing songs. For each activity in each language, parents selected one of three response options: 0 = rarely or never, 1 = at least once a week, and 2 = daily. Higher scores indicated more frequent engagement in home literacy activities in the corresponding language. Consistent with the home biliteracy model and the study’s focus on language-specific and activity-specific associations with receptive vocabulary, each home literacy activity was analyzed separately for English and Chinese.

### 2.4. Bilingual Receptive Vocabulary

#### 2.4.1. English Receptive Vocabulary

Children’s English receptive vocabulary was assessed using the Peabody Picture Vocabulary Test, 5th edition (PPVT-5; D. M. Dunn, 2019). The PPVT-5 is an individually administered measure of receptive vocabulary designed for individuals aged 2 years 6 months and older. It has been widely used in studies of bilingual children’s vocabulary development (e.g., Ji et al., 2022; Li et al., 2021; Uchikoshi, 2013). The test consists of 240 items arranged in order of increasing difficulty and typically takes approximately 10 to 15 min to administer. The PPVT-5 manual reports high overall reliability for the instrument, with a reliability coefficient of 0.97 (D. M. Dunn, 2019). In the present study, trained research assistants administered the test individually to each child in community libraries or in participants’ homes. For each item, the examiner pronounced a target word and presented four pictures, and the child was asked to point to the picture that best represented the word. Testing was discontinued after six consecutive incorrect responses. Raw scores were converted to age-normed standard scores (Mean = 100; *SD* = 15) using the tables provided in the PPVT-5 manual.

#### 2.4.2. Chinese Receptive Vocabulary

Children’s Chinese receptive vocabulary was assessed using the Chinese Peabody Picture Vocabulary Test-Revised, Form M (C-PPVT-R; Lu & Liu, 2005). The C-PPVT-R is an adaptation of the English PPVT-R (L. M. Dunn & Dunn, 1981) and is designed to assess receptive vocabulary among children aged 3 to 16. The test includes 125 items presented in increasing order of difficulty and has been used in previous studies as a reliable measure of Chinese receptive vocabulary among bilingual children. In this study, trained multilingual research assistants administered the test individually in the child’s heritage language, either Mandarin or Cantonese, depending on the child’s home language background. As in the English PPVT, each item required the child to select one picture from four options that best matched the spoken word. Administration began with age-appropriate items and was discontinued after six consecutive incorrect responses. Each correct response received one point, and raw scores were converted to age-normed standard scores (Mean = 100; *SD* = 15) based on the test manual. These standard scores, which allowed us to compare children’s receptive vocabulary performance with age-based norms in each language, were used in the final analyses.

### 2.5. Data Collection

Data were collected during the 2021–2022 and 2022–2023 school years. In each school year, parents completed the demographic and home literacy activity questionnaire through an online platform. The questionnaire was administered one-on-one in the language chosen by the parents, either English, Mandarin, or Cantonese, and took approximately one hour to complete. The home literacy activity data used in the present study were drawn from this larger questionnaire. Children’s receptive vocabulary was assessed in accordance with the administration manuals for the respective tests. Assessments were conducted either online or in person at participants’ homes or local libraries, depending on family preference. These tests were not timed and following the manual instructions, administration was discontinued after a child made six consecutive errors. Administration time therefore varied considerably across children as children who obtained higher scores generally completed more items and required more time.

### 2.6. Data Analysis

Descriptive statistics were first computed to summarize children’s English and Chinese receptive vocabulary scores and the frequency of home literacy activities in each language. Means and standard deviations were calculated for continuous variables, and frequencies and percentages were calculated for demographic variables, including gender, family SES, and immigration status. Correlation analyses were then conducted to examine bivariate associations among receptive vocabulary scores, home literacy activities, and demographic variables. These analyses provided an initial overview of the relationships between English and Chinese home literacy activities and children’s receptive vocabulary in each language.

Hierarchical linear regression analyses were conducted to examine whether home literacy activities were associated with children’s receptive vocabulary after controlling for demographic variables. Separate models were estimated by grade level and language outcome. For each model, demographic variables were entered in Step 1, including age, gender, family SES, and immigration status. Home literacy activities in the corresponding language were entered in Step 2. Specifically, English home literacy activities were included in the model for English receptive vocabulary, and Chinese home literacy activities were included in the model for Chinese receptive vocabulary.

Gender, family SES, and immigration status were dummy-coded as follows: gender was coded as 0 = male and 1 = female; family SES was coded as 0 = low-SES and 1 = non-low-SES; and immigration status was coded as 0 = immigrant and 1 = Canadian-born. Cases with missing values on variables included in each model were excluded using listwise deletion. Prior to interpreting the regression results, model assumptions were examined, including linearity, normality of residuals, and multicollinearity. All analyses were conducted using SPSS version 28.0.

## 3. Results

### 3.1. Patterns of Bilingual Home Literacy Activities and Receptive Vocabulary

To address the first research question (i.e., what are the patterns of English and Chinese home literacy activities and receptive vocabulary among Chinese–English bilingual children in Grade 1 and Grade 2?), we examined the patterns of English and Chinese home literacy activities and receptive vocabulary among children in Grade 1 and Grade 2. As shown in Table 3, children engaged more frequently in English than Chinese home literacy activities across both grades. Book or magazine reading in English was reported at a relatively high frequency in both Grade 1 (*M* = 1.72, *SD* = 0.55) and Grade 2 (*M* = 1.82, *SD* = 0.46), whereas Chinese book or magazine reading was less frequent and remained stable across grades (Grade 1: *M* = 0.85, *SD* = 0.79; Grade 2: *M* = 0.85, *SD* = 0.75). Similar patterns were observed for computer or laptop use, television or movie watching, and singing songs, all of which occurred more frequently in English than in Chinese.

Storytelling showed a smaller language difference than the other activities. For the first graders, the mean frequency of storytelling was 1.22 (*SD* = 0.78) in English and 1.04 (*SD* = 0.77) in Chinese. For the second graders, the corresponding means were 1.13 (*SD* = 0.80) and 0.99 (*SD* = 0.83). In comparing the two grade-level samples, the mean frequencies of English book or magazine reading and English computer or laptop use were slightly higher in Grade 2 than in Grade 1, whereas English storytelling and singing songs were slightly less frequent in Grade 2. Chinese home literacy activities were relatively stable across samples, except for Chinese computer or laptop use, which was less frequent in Grade 1 (*M* = 0.47, *SD* = 0.63) than in Grade 2 (*M* = 0.62, *SD* = 0.75). These descriptive results suggest that English home literacy activities were more frequent overall, while Chinese home literacy activities were less frequent and more concentrated in storytelling and book or magazine reading.

Table 4 presents descriptive statistics for children’s English and Chinese receptive vocabulary scores by grade level, gender, family socioeconomic status (SES), and immigration status. In terms of grade-level differences, both English and Chinese receptive vocabulary scores were higher, on average, in the Grade 2 sample than in the Grade 1 sample. The mean PPVT-English score was 90.90 (*SD* = 14.44) in Grade 1 and 96.94 (*SD* = 15.31) in Grade 2. Similarly, the mean PPVT-Chinese score was 94.05 (*SD* = 17.31) in Grade 1 and 100.70 (*SD* = 22.86) in Grade 2.

Gender differences were relatively small. In Grade 1, girls had a higher mean PPVT-English score (*M* = 93.54, *SD* = 14.13) than boys (*M* = 88.13, *SD* = 14.35). In Grade 2, the gender gap was smaller, with girls scoring only 0.63 points higher than boys on average. For Chinese receptive vocabulary, boys and girls had nearly identical mean scores in both grades.

Differences by family socioeconomic status (SES) were more evident for English than for Chinese receptive vocabulary. In both grades, children from non-low-SES families had higher mean PPVT-English scores than those from low-SES families. In Grade 1, the mean PPVT-English score was 92.23 (*SD* = 13.12) for children from non-low-SES families and 86.39 (*SD* = 17.77) for those from low-SES families. In Grade 2, the corresponding means were 98.83 (*SD* = 14.26) and 91.53 (*SD* = 17.11). By contrast, no comparable SES pattern was observed for Chinese receptive vocabulary; mean PPVT-Chinese scores were similar across SES groups, with children from low-SES families showing slightly higher average scores in both grades.

A different pattern was observed for immigration status. English receptive vocabulary scores were similar for immigrant and Canadian-born children in Grade 1, but Canadian-born children had a higher mean PPVT-English score than immigrant children in Grade 2. In contrast, immigrant children had higher mean PPVT-Chinese scores than Canadian-born children in both grades. In Grade 1, immigrant children had a mean PPVT-Chinese score of 100.33 (*SD* = 16.61), compared with 92.17 (*SD* = 17.15) for Canadian-born children. In Grade 2, the corresponding means were 107.26 (*SD* = 17.48) and 98.27 (*SD* = 24.19). These descriptive patterns suggest that English receptive vocabulary was more strongly differentiated by family SES, whereas Chinese receptive vocabulary appeared to be more closely related to immigration background.

### 3.2. Within- and Cross-Language Bivariate Associations Between Bilingual Home Literacy Activities and Receptive Vocabulary

For the second research question (i.e., what within- and cross-language bivariate associations exist between English and Chinese home literacy activities and children’s English and Chinese receptive vocabulary in Grades 1 and 2?), Spearman rank-order correlations were computed separately for Grade 1 and Grade 2. As shown in Table 5, in Grade 1, English receptive vocabulary was positively associated with age (ρ = 0.19, *p* < 0.05), gender (ρ = 0.21, *p* < 0.05), family socioeconomic status (SES; ρ = 0.27, *p* < 0.01), and English book or magazine reading (ρ = 0.27, *p* < 0.01). None of the other English home literacy activities were significantly associated with English receptive vocabulary. In contrast, Chinese receptive vocabulary showed a different pattern. It was negatively associated with immigration status (ρ = −0.22, *p* < 0.05), indicating higher Chinese receptive vocabulary among immigrant children than among Canadian-born children. Chinese receptive vocabulary was also positively associated with several Chinese home literacy activities, including book or magazine reading (ρ = 0.29, *p* < 0.01), computer or laptop use (ρ = 0.22, *p* < 0.05), television or movie watching (ρ = 0.22, *p* < 0.05), and storytelling (ρ = 0.26, *p* < 0.01). 

In Grade 2 (Table 6), English receptive vocabulary was positively correlated with family SES (*ρ* = 0.26, *p* < 0.01) and English book or magazine reading (*ρ* = 0.18, *p* < 0.05). It was negatively correlated with Chinese computer or laptop use (*ρ* = −0.18, *p* < 0.05), although the association was small. Chinese receptive vocabulary was positively correlated with age (*ρ* = 0.21, *p* < 0.05) and several Chinese home literacy activities, including Chinese book or magazine reading (*ρ* = 0.43, *p* < 0.01), Chinese computer or laptop use (ρ = 0.34, *p* < 0.01), and Chinese storytelling (*ρ* = 0.36, *p* < 0.01). Chinese receptive vocabulary was also positively correlated with English computer or laptop use (*ρ* = 0.20, *p* < 0.05) and English storytelling (*ρ* = 0.21, *p* < 0.05).

Overall, the correlation results showed that English receptive vocabulary was associated with a limited set of variables, mainly family SES and English book or magazine reading. In contrast, Chinese receptive vocabulary was associated with a broader range of Chinese home literacy activities in both grades. This pattern provides evidence that Chinese receptive vocabulary is more closely related to home-based literacy activities than English receptive vocabulary among the bilingual children.

### 3.3. Association Between Language-Specific Home Literacy Activities and Receptive Vocabulary

Finally, to address the third research question (i.e., after controlling for demographic characteristics, which language-specific home literacy activities are uniquely associated with children’s receptive vocabulary in the corresponding language in Grades 1 and 2?), hierarchical linear regression analyses were conducted separately by grade level and language. As shown in Table 7, for Grade 1 English receptive vocabulary, the demographic variables entered in Step 1 explained 13% of the variance (*R*^2^ = 0.13), *F* = 4.20, *p* < 0.01. Age (β = 0.24, *p* < 0.01), gender (β = 0.21, *p* < 0.05), and family socioeconomic status (SES; β = 0.20, *p* < 0.05) were significant predictors, indicating that older children, girls, and children from non-low-SES families tended to have higher English receptive vocabulary scores. After the English home literacy activities were entered in Step 2, the model explained 19% of the variance. However, the additional variance explained by the home literacy activities was not statistically significant (Δ*R*^2^ = 0.07, *F* change = 1.82, *p* > 0.05). Among the English home literacy activities, only singing songs was a significant predictor of English receptive vocabulary (β = 0.19, *p* < 0.05).

For Chinese receptive vocabulary, demographic variables entered in Step 1 explained 5% of the variance, *R*^2^ = 0.05, F = 1.35, *p* > 0.05. Immigration status was a significant variable in Step 1 (*β* = −0.21, *p* < 0.05), indicating that immigrant children tended to have higher Chinese receptive vocabulary scores than Canadian-born children. After Chinese home literacy activities were added in Step 2, the model explained 19% of the variance. The addition of Chinese home literacy activities significantly improved the model, Δ*R*^2^ = 0.15, *F* change = 3.89, *p* < 0.01. Among the Chinese home literacy activities, reading books or magazines was the only significant variable for Chinese receptive vocabulary (*β* = 0.20, *p* < 0.05). Overall, the Grade 1 results suggest that, compared with English receptive vocabulary, Chinese receptive vocabulary showed a stronger link to home literacy activities, especially Chinese book or magazine reading.

In Grade 2 (Table 8), demographic variables entered in Step 1 explained 9% of the variance for English receptive vocabulary: *R*^2^ = 0.09, *F* = 2.91, *p* < 0.05. Family SES was the only significant demographic variable (*β* = 0.20, *p* < 0.05), indicating that children from non-low-SES families tended to have higher English receptive vocabulary scores. After English home literacy activities were added in Step 2, the model explained 13% of the variance. However, the increase in explained variance was not statistically significant, Δ*R*^2^ = 0.04, *F* change = 1.17, *p* > 0.05. Among the English home literacy activities, reading books or magazines was the only significant variable for English receptive vocabulary (*β* = 0.19, *p* < 0.05).

For Chinese receptive vocabulary, demographic variables entered in Step 1 explained 9% of the variance, *R*^2^ = 0.09, *F* = 2.81, *p* < 0.05. Age (*β* = 0.22, *p* < 0.05) and immigration status (*β* = −0.19, *p* < 0.05) were significant variables, indicating that older children and immigrant children tended to have higher Chinese receptive vocabulary scores. After Chinese home literacy activities were added in Step 2, the model explained an extra 23% of the variance. The addition of Chinese home literacy activities significantly improved the model: Δ*R*^2^ = 0.23, *F* change = 7.70, *p* < 0.01. Among the Chinese home literacy activities, reading books or magazines (*β* = 0.23, *p* < 0.05), using a computer or laptop (*β* = 0.23, *p* < 0.05), and storytelling (*β* = 0.22, *p* < 0.05) were significant variables of Chinese receptive vocabulary. In summary, the Grade 2 results provide even clearer evidence for stronger HLE-based associations of Chinese receptive vocabulary than in Grade 1: after demographic variables were controlled, Chinese home literacy activities significantly increased the explained variance, and the full model accounted for 32% of the variance in Chinese receptive vocabulary.

## 4. Discussion

The present study examined how English and Chinese home literacy activities were associated with English and Chinese receptive vocabulary among Chinese–English bilingual children in Grade 1 and Grade 2. Building on the home biliteracy model proposed by Li et al. (2025), this study provides further evidence that a bilingual home literacy environment (HLE) should be understood not only as language-specific, but also as activity-specific. Three major findings are worth highlighting. First, English home literacy activities were more frequent than Chinese home literacy activities, and the activity profiles in both languages differed across the two grade levels. Second, although English home literacy activities were more frequent, Chinese receptive vocabulary was more strongly associated with Chinese home literacy activities. Third, English and Chinese receptive vocabulary appeared to be associated with different kinds of home literacy activities, suggesting that heritage-language vocabulary may require broader home-based support than mainstream-language vocabulary. A discussion of the findings follows.

To begin with, the descriptive statistics showed that English home literacy activities were generally more frequent than Chinese home literacy activities across both grades. This pattern is consistent with previous research showing that bilingual children in English-dominant contexts often have greater access to English literacy resources and English-mediated activities than to heritage-language resources and activities (Duursma et al., 2007; Hoff, 2018; Li et al., 2025, 2026; Ryan, 2021). In the present study, various English literacy activities, including book or magazine reading, computer or laptop use, television or movie watching, and singing songs, were all reported more frequently than the corresponding activities in Chinese. At the same time, the descriptive results also showed that book or magazine reading and computer or laptop use were reported more frequently among Grade 2 children than among Grade 1 children, whereas storytelling and singing songs were reported slightly less frequently among the second graders.

These patterns suggest that home literacy activities may become more print- and digitally oriented as children move through the early elementary grades. Book reading may become more frequent as children’s literacy development is more closely aligned with school-based literacy expectations, while computer or laptop use may become more common as children gain access to digital learning resources and literacy-related technologies (e.g., Dore et al., 2020; Li et al., 2024). In contrast, oral and routine-based activities, such as storytelling and singing songs, may become less frequent as children grow older and as home literacy practices shift toward more independent forms of literacy engagement. These patterns are consistent with existing research showing that home literacy activity profiles may shift during the early elementary years (Li et al., 2026; Silinskas et al., 2020). Such changes may also reflect children’s developing literacy skills and growing independence, which enable them to engage in book reading and computer-based literacy activities with less direct adult mediation.

Second, although English home literacy activities were more frequent overall, English receptive vocabulary was less strongly associated with English home literacy activities after demographic variables were controlled. In contrast, Chinese home literacy activities were more strongly associated with Chinese receptive vocabulary in both grades. In our analyses, adding Chinese home literacy activities accounted for an additional 15% and 23% of the variance in Chinese receptive vocabulary in Grade 1 and Grade 2, respectively. This pattern provides further evidence for the language-specific effects of HLE in bilingual families (Goodrich et al., 2021; Li et al., 2025; Ryan, 2021). Previous bilingual HLE studies have shown that home literacy practices are not associated with bilingual vocabulary development in the same way across languages (e.g., Goodrich et al., 2021; Li et al., 2025; Ryan, 2021). The present study extends this pattern into Grade 1 and Grade 2, showing that the stronger HLE-based associations of heritage-language vocabulary continue beyond the first year of formal schooling.

Based on the findings, one possible explanation is that, as children move through English-medium schooling, English vocabulary development becomes increasingly supported by formal schooling and the wider English-speaking environment (Duursma et al., 2007; Hoff, 2018; Paradis, 2011). As a result, the unique contribution of English home literacy activities may become less salient when other sources of English exposure are considered. Chinese vocabulary development, however, remains more closely associated with home-based literacy activities because Chinese input is less consistently available in school and community contexts (e.g., Cheung et al., 2019; Li et al., 2025, 2026; Sun et al., 2024). In this sense, the significance of HLE cannot be evaluated not only by the frequency or quality of home literacy activities, but also within a wider social context in which they occur. Unequal access to language and literacy resources across home, school, and community means that similar levels of home literacy activity may carry different developmental weight in different contexts.

Third, the findings suggest that English and Chinese receptive vocabulary were associated with different types of literacy activities at home. English receptive vocabulary was linked to a relatively narrow set of activities: English singing songs in Grade 1 and English book or magazine reading in Grade 2. This pattern is broadly consistent with studies highlighting the role of print resources and children’s independent literacy engagement in HLE (Georgiou et al., 2021; Silinskas et al., 2020; van Bergen et al., 2017). In contrast, Chinese receptive vocabulary showed a broader association with home literacy activities. In Grade 1, Chinese book or magazine reading was the only significant home literacy variable. By Grade 2, however, Chinese book or magazine reading, Chinese computer or laptop use, and Chinese storytelling were all significantly associated with Chinese receptive vocabulary. These findings suggest that Chinese receptive vocabulary may be linked to multiple forms of home support, including print-based exposure, digital resources, and parent–child interactions.

The activity-level findings further illustrate the multidimensional nature of bilingual HLE conceptualized in the home biliteracy model (Li et al., 2025), which encompasses not only parent-engaged literacy activities, such as shared reading and direct teaching, but also print resources, digital access, and child-independent literacy activities. Although the present study does not directly measure the degree of adult support involved in each activity, the findings extend this framework by demonstrating the value of examining specific literacy activities within each language, rather than treating HLE as a single or undifferentiated construct. They further suggest that the dimensions of HLE associated with vocabulary development may differ by language and developmental stage.

The broad range of associations observed for heritage-language vocabulary development may reflect the particular role of the home in supporting heritage-language development. Book or magazine reading may provide access to print-based and contextualized vocabulary in Chinese; computer or laptop use may offer additional Chinese input through digital media and online resources; and storytelling may create opportunities for oral language interactions. Because opportunities for Chinese language and literacy engagement may be more limited outside the home, these different activities may provide complementary sources of heritage-language input. As bilingual children move further into mainstream schooling, maintaining a diverse set of home-based literacy opportunities may therefore become particularly important for heritage-language development.

## 5. Limitations and Implications

While the findings of the present study add to current understandings about home literacy activities among bilingual families, several limitations should be noted. First, although the data were drawn from a larger longitudinal project, the present study used a repeated cross-sectional design and therefore cannot directly capture developmental changes over time. Future studies using a longitudinal design to follow the same cohort of participants are needed. Second, in this study, home literacy activities were measured through parent reports using a three-point frequency scale. While this measure provided a broader indication of how often children engaged in different literacy activities, it could not fully capture the quality and duration of these experiences. Future research should use more detailed measures, such as parent interviews or observation notes, to better capture the quality and content of the home literacy practices. Third, the study focused on Chinese–English bilingual children in one Canadian metropolitan context and measured receptive vocabulary only, so future research could examine whether similar patterns are found in other bilingual populations and across broader language and literacy outcomes.

Despite these limitations, the study has several research and practical implications. Theoretically, the findings support the need to understand bilingual HLE as both language-specific and activity-specific. English home literacy activities were more frequent, but Chinese home literacy activities showed stronger and broader associations with Chinese receptive vocabulary. This suggests that the developmental significance of HLE cannot be inferred from literacy activities or resources at home alone; it also depends on how each language is differently supported, valued, and made available across home, school, and community. Practically, the findings indicate that heritage-language development may benefit from diverse home-based literacy opportunities, including print-based, digital, and oral interactions. For parents, this means that supporting bilingual development requires attention not only to how often children engage in literacy activities, but also to which language these activities occur in and what kinds of language exposure they provide.

## 6. Conclusions

In this study, we examined the associations between English and Chinese home literacy activities and bilingual receptive vocabulary among children in Grade 1 and Grade 2. The findings showed that, among the bilingual families, English home literacy activities were generally more frequent than Chinese home literacy activities. At the same time, the literacy activities at home may gradually become more print- and digitally oriented as children move through the early elementary grades. More importantly, although English home literacy activities were more frequent overall, Chinese receptive vocabulary was more strongly and more broadly associated with Chinese home literacy activities. In particular, Chinese book or magazine reading was associated with Chinese receptive vocabulary in both grades, while Chinese computer or laptop use and storytelling also became significant in Grade 2. These findings suggest that understanding bilingual HLE should go beyond how much or how frequently children experience literacy activities in each language at home. Instead, it requires attention to how families organize literacy opportunities across languages within a wider and often unequal language environment. For Chinese–English bilingual children in an English-dominant context, as this study shows, the home remains an especially critical space for sustaining Chinese language and literacy development as children move through the early elementary years.

## Figures and Tables

**Table 1:** Demographic information of participants.

	Grade 1				Grade 2			
	Mean	*SD*	*N*	%	Mean	*SD*	*N*	%
Age (in months)	78.15	3.99			89.51	4.61		
Gender								
Male	-	-	60	48.78%	-	-	64	50.79%
Female	-	-	63	51.22%	-	-	62	49.21%
Family SES								
Low-SES	-	-	28	22.76%	-	-	33	26.19%
Non-low-SES	-	-	95	77.24%	-	-	93	73.81%
Immigration status								
Immigrants	-	-	27	21.95%	-	-	34	26.98%
Born in Canada	-	-	96	78.05%	-	-	92	73.02%
Total	-	-	123	100%	-	-	126	100%

**Table 2:** Bilingual home literacy activity questionnaire.

	In English			In Chinese		
	Daily	At Least Once a Week	Rarely or Never	Daily	At Least Once a Week	Rarely or Never
Reading books or magazines	2	1	0	2	1	0
Using a computer or laptop	2	1	0	2	1	0
Watching television or movies	2	1	0	2	1	0
Storytelling	2	1	0	2	1	0
Singing songs	2	1	0	2	1	0

**Table 3:** Descriptive statistics of bilingual home literacy activities questionnaire.

Home Literacy Activities	English				Chinese			
	Grade 1		Grade 2		Grade 1		Grade 2	
	Mean	*SD*	Mean	*SD*	Mean	*SD*	Mean	*SD*
Reading books or magazines	1.72	0.55	1.82	0.46	0.85	0.79	0.85	0.75
Using a computer or laptop	1.36	0.79	1.57	0.64	0.47	0.63	0.62	0.75
Watching television or movies	1.20	0.75	1.20	0.68	0.66	0.68	0.63	0.71
Storytelling	1.22	0.78	1.13	0.80	1.04	0.77	0.99	0.83
Singing songs	1.11	0.75	0.98	0.82	0.43	0.62	0.41	0.66

**Table 4:** Descriptive statistics of bilingual receptive vocabulary scores.

	PPVT-English				PPVT-Chinese			
	Grade 1		Grade 2		Grade 1		Grade 2	
	Mean	*SD*	Mean	*SD*	Mean	*SD*	Mean	*SD*
Total	90.90	14.44	96.94	15.31	94.05	17.31	100.70	22.86
Gender								
Male	88.13	14.35	96.63	17.68	94.07	17.41	100.45	26.01
Female	93.54	14.13	97.26	12.55	94.03	17.36	100.95	19.30
Family SES								
Low-SES	86.39	17.77	91.53	17.11	98.26	19.73	102.42	20.59
Non-low-SES	92.23	13.12	98.83	14.26	92.79	16.42	100.09	23.69
Immigration status								
Immigrants	90.56	21.61	92.85	15.92	100.33	16.61	107.26	17.48
Born in Canada	91.00	11.82	98.43	14.90	92.17	17.15	98.27	24.19

**Table 5:** Spearman correlations among demographic variables, home literacy activities, and receptive vocabulary in grade 1.

		1	2	3	4	5	6	7	8	9	10	11	12	13	14	15	16
1	Age	—															
2	Gender	−0.09	—														
3	SES	0.00	0.059	—													
4	Immigration Status	−0.04	0.22 *	0.08	—												
5	Reading books or magazines in English	0.12	0.07	0.13	−0.17	—											
6	Using a computer or laptop in English	0.10	0.07	0.14	−0.01	−0.07	—										
7	Watching television or movies in English	0.25 **	−0.07	0.16	−0.14	0.08	0.30 **	—									
8	Storytelling in English	0.02	−0.22 *	−0.08	−0.08	0.18	0.17	0.21 *	—								
9	Singing songs in English	−0.07	0.14	0.03	0.03	0.18	−0.07	0.09	0.33 **	—							
10	Reading books or magazines in Chinese	−0.16	−0.03	−0.03	−0.08	0.02	−0.14	−0.23 *	0.02	−0.09	—						
11	Using a computer or laptop in Chinese	−0.01	0.03	0.00	−0.14	−0.05	0.22 *	−0.03	0.00	−0.14	0.21 *	—					
12	Watching television or movies in Chinese	−0.01	0.13	0.00	0.01	−0.03	0.16	0.11	0.19 *	−0.09	0.13	0.25 **	—				
13	Storytelling in Chinese	0.03	−0.11	0.00	0.01	0.08	0.11	−0.05	0.16	0.02	0.39 **	0.21 *	0.18 *	—			
14	Singing songs in Chinese	−0.05	0.08	0.02	−0.10	−0.03	0.02	0.04	0.02	0.23 *	0.15	0.33 **	0.27 **	0.19 *	—		
15	English receptive vocabulary	0.19 *	0.21 *	0.27 **	0.08	0.27 **	0.06	0.17	0.00	0.17	−0.17	−0.07	0.00	−0.11	−0.02	—	
16	Chinese receptive vocabulary	−0.04	−0.02	−0.12	−0.22 *	−0.06	−0.04	−0.16	−0.03	−0.13	0.29 **	0.22 *	0.22 *	0.26 **	0.11	−0.01	—

Note. Values are Spearman’s ρ. All tests are two-tailed. Boldface indicates statistically significant correlations: * *p* < 0.05. ** *p* < 0.01.

**Table 6:** Spearman correlations among demographic variables, home literacy activities, and receptive vocabulary in grade 2.

		1	2	3	4	5	6	7	8	9	10	11	12	13	14	15	16
1	Age	—															
2	Gender	−0.16	—														
3	SES	−0.02	0.15	—													
4	Immigration Status	−0.09	0.24 **	0.09	—												
5	Reading books or magazines in English	0.07	−0.04	0.05	−0.06	—											
6	Using a computer or laptop in English	0.14	−0.08	−0.06	−0.21 *	0.05	—										
7	Watching television or movies in English	0.11	−0.15	−0.09	−0.17	0.06	0.38 **	—									
8	Storytelling in English	−0.02	0.11	−0.06	0.05	0.16	0.27 **	0.25 **	—								
9	Singing songs in English	−0.12	0.20 *	0.03	−0.01	0.08	0.03	0.25 **	0.32 **	—							
10	Reading books or magazines in Chinese	−0.09	−0.01	0.12	−0.12	0.18 *	0.12	−0.13	0.02	−0.06	—						
11	Using a computer or laptop in Chinese	−0.09	0.03	0.03	−0.13	−0.03	**0.1** **8** *	0.11	0.20 *	0.03	0.48 **	—					
12	Watching television or movies in Chinese	0.01	−0.07	0.09	−0.09	0.01	0.24 **	0.22 *	0.10	−0.03	0.23 *	0.50 **	—				
13	Storytelling in Chinese	0.00	0.03	−0.09	−0.01	0.30 **	0.06	0.04	0.34 **	0.14	0.38 **	0.27 **	0.15	—			
14	Singing songs in Chinese	−0.13	0.09	−0.03	−0.02	−0.08	0.05	0.05	0.01	0.21 *	0.17	0.29 **	0.19 *	0.03	—		
15	English receptive vocabulary	0.16	0.08	0.26 **	0.16	0.18 *	0.03	0.04	0.11	0.05	0.03	−0.18 *	−0.04	0.00	−0.12	—	
16	Chinese receptive vocabulary	0.21 *	−0.01	−0.05	−0.14	0.04	0.20 *	−0.15	0.21 *	−0.08	0.43 **	0.34 **	0.09	0.36 **	0.10	0.03	—

Note. Values are Spearman’s ρ. All tests are two-tailed. Boldface indicates statistically significant correlations: * *p* < 0.05. ** *p* < 0.01.

**Table 7:** Hierarchical linear regression for English and Chinese receptive vocabulary in grade 1.

	Model 1		Model 2	
Variables	PPVT-English	PPVT-Chinese	PPVT-English	PPVT-Chinese
Step 1. Demographic variables				
Age	0.24 **	−0.05	0.23 *	−0.02
Gender	0.21 *	0.03	0.17	0.04
SES	0.20 *	0.03	0.20 *	−0.01
Immigration status	−0.06	−0.21 *	−0.03	−0.18
* R* ^2^	0.13	0.05	0.13	0.05
* F*	4.20 **	1.35	4.20 **	1.35
Step 2. Home literacy activities				
Reading books or magazines			0.12	0.20 *
Using a computer or laptop			−0.04	0.16
Watching television or movies			0.10	0.09
Storytelling			−0.05	0.14
Singing songs			0.19 *	−0.01
* R* ^2^			0.19	0.19
* *Δ*R*^2^			0.07	0.15
* F* change			1.82	3.89 **

Note. Values are standardized regression coefficients (*β*). Boldface indicates statistically significant correlations: * *p* < 0.05. ** *p* < 0.01.

**Table 8:** Hierarchical linear regression for English and Chinese receptive vocabulary in grade 2.

	Model 1		Model 2	
Variables	PPVT-English	PPVT-Chinese	PPVT-English	PPVT-Chinese
Step 1. Demographic variables				
Age	0.17	0.22 *	0.14	0.23 **
Gender	−0.03	0.08	−0.03	0.05
SES	0.20 *	0.06	0.20 *	0.04
Immigration status	0.15	−0.19 *	0.15	−0.13
* R* ^2^	0.09	0.09	0.09	0.09
* F*	2.91 *	2.81 *	2.91 *	2.81 *
Step 2. Home literacy activities				
Reading books or magazines			0.19 *	0.23 *
Using a computer or laptop			0.03	0.23 *
Watching television or movies			−0.02	−0.16
Storytelling			0.07	0.22 *
Singing songs			−0.02	0.02
* R* ^2^			0.13	0.32
* *Δ*R*^2^			0.04	0.23
* F* change			1.17	7.70 **

Note. Values are standardized regression coefficients (*β*). Boldface indicates statistically significant correlations: * *p* < 0.05. ** *p* < 0.01.

## Data Availability

The original data presented in the study are openly available in the Open Science Framework at https://osf.io/wta7k/files/n9g5z?view_only=0167764bffc34686a8253796cd49e263 (accessed on 6 July 2026).
